# 

**Published:** 1899-11

**Authors:** 


CONTENTS.

ORIGINAL ARTICLES:
- Meat-inspection in Small Cities and Towns. By JOHN J. REPP, V.M.D. . . . . . 671
- Disinfection. By E. A. A. GRANGE, M.D., V.S. . . . . . . . . . . 674
- A Plea for the More General Use of Anæsthesia in Veterinary Surgery. By J. P. TURNER, V.M.D. . . . . . . 681
- Clinical Lectures. By S. J. J. HARGER, V.M.D. . . . . . . . . . 686
- European Veterinary Institutions. By JAS. B. PAIGE, D.V.S. . . . . . . . 689
- Tetanus Toxin and Antitoxin. By JOSEPH McFARLAND, M.D., and E. M. RANCK, V.M.D. . . . . . . . . . 694
- Dietetics. By W.H.DALRYMPLE, M.R.C.V.S. 699

SELECTION:
- Inoculation Against Texas Fever . . . 705

COMMUNICATION . . . . . . . . . 709

THERAPEUTICAL AND PHARMACEUTICAL NOTES 710

DEPARTMENT OF SURGERY:

- Quittor . . . . . . . . . . 717

REPORTS OF CASES:
- Myoneurectomy for Cribbing. By S. J. J. HARGER, V.M.D. . . . . . . 720

EDITORIAL:
- Where in 1900? . . . . . . . 723

NECROLOGY . . . . . . . . . . 725

THE STORY OF NEW YORK BRIEFLY TOLD . 728

SOCIETY PROCEEDINGS:
- Missouri Valley — Minnesota — Chicago — New Jersey . . . . . . . . 730

AMONG THE COLLEGES . . . . . 733

PERSONALS . . . . . . . . . . 733

